# Centromeric signaling proteins boost G1 cyclin degradation and modulate cell size in budding yeast

**DOI:** 10.1371/journal.pbio.2005388

**Published:** 2018-08-06

**Authors:** Joan M. Martínez-Láinez, David F. Moreno, Eva Parisi, Josep Clotet, Martí Aldea

**Affiliations:** 1 Institut de Biologia Molecular de Barcelona IBMB-CSIC, Barcelona, Catalonia, Spain; 2 Departament de Ciències Bàsiques, Universitat Internacional de Catalunya, Barcelona, Spain; The Institute of Cancer Research, United Kingdom of Great Britain and Northern Ireland

## Abstract

Cell size scales with ploidy in a great range of eukaryotes, but the underlying mechanisms remain unknown. Using various orthogonal single-cell approaches, we show that cell size increases linearly with centromere (CEN) copy number in budding yeast. This effect is due to a G1 delay mediated by increased degradation of Cln3, the most upstream G1 cyclin acting at Start, and specific centromeric signaling proteins, namely Mad3 and Bub3. Mad3 binds both Cln3 and Cdc4, the adaptor component of the Skp1/Cul1/F-box (SCF) complex that targets Cln3 for degradation, these interactions being essential for the CEN-dosage dependent effects on cell size. Our results reveal a pathway that modulates cell size as a function of CEN number, and we speculate that, in cooperation with other CEN-independent mechanisms, it could assist the cell to attain efficient mass/ploidy ratios.

## Author summary

It has been known for a long time that cells increase their size with the amount of DNA across the whole evolutionary scale. Moreover, within a given species and differentiation status, cells increase their size in an exquisitely proportional manner to the number of chromosomes. This correlation has been observed in fungi, plants, and animals, and it is assumed to have important implications in development and physiology. However, the molecular mechanisms that set cell size as a function of ploidy are still a mystery. Here, we uncover a pathway that links the number of chromosomes to the molecular network that triggers cell cycle entry in budding yeast. More specifically, we have found that an excess number of centromeres (CENs) increases degradation of Cln3, a G1 cyclin that plays a key role at Start, thus delaying entry into the cell cycle and, as cell growth is not affected, increasing cell size. Our results point to the existence of dedicated mechanisms operating during entry into the cell cycle to adjust cell size to ploidy.

## Introduction

Most cells under unaltered conditions of growth are able to maintain their size within a strict range, and a current view sustains that the cell cycle and cell growth machineries should be interconnected by specific molecular mechanisms ensuring cell size homeostasis [1–5]. Budding yeast cells control their size mainly at Start [6,7], when a G1 cyclin, Cln3, acts as the most upstream activator [8]. Cyclin Cln3 forms a complex with Cdc28, the cell cycle Cdk in budding yeast, which phosphorylates the transcriptional inhibitor Whi5 and induces a transcriptional wave in circa 200 genes to trigger cell cycle entry [9]. Cln3 modulates cell volume at Start in a precise, dose-dependent manner [10–12], which suggests that mechanisms regulating its levels or activity likely play important roles in cell size determination. In this regard, Cln3 is present at low and nearly constant amounts throughout G1 [8, but see 13,14], and its nuclear levels are restrained by retention at the ER [15,16] and ubiquitin-mediated degradation by the proteasome [17,18].

It has long been known that cell size increases linearly with ploidy in fungi [19–21], plants [22], and animals [23,24], a function that is maintained across the enormous DNA content variation among eukaryotes [25] and has been used to infer ploidy in the fossil record [26]. Although ploidy has direct implications in cell growth and development, the underlying mechanisms that set cell size as a function of ploidy remain elusive [27]. Here, we describe a pathway linking the centromere (CEN) to the Start network in budding yeast. Briefly, we have found that an excess number of CENs increases degradation of Cln3 in the nucleus by a mechanism that involves physical and functional interactions between Cdc4, the specific F-box protein that targets Cln3 to SCF for ubiquitination, and Mad3, a centromeric signaling protein.

## Results and discussion

**Fig 1:**
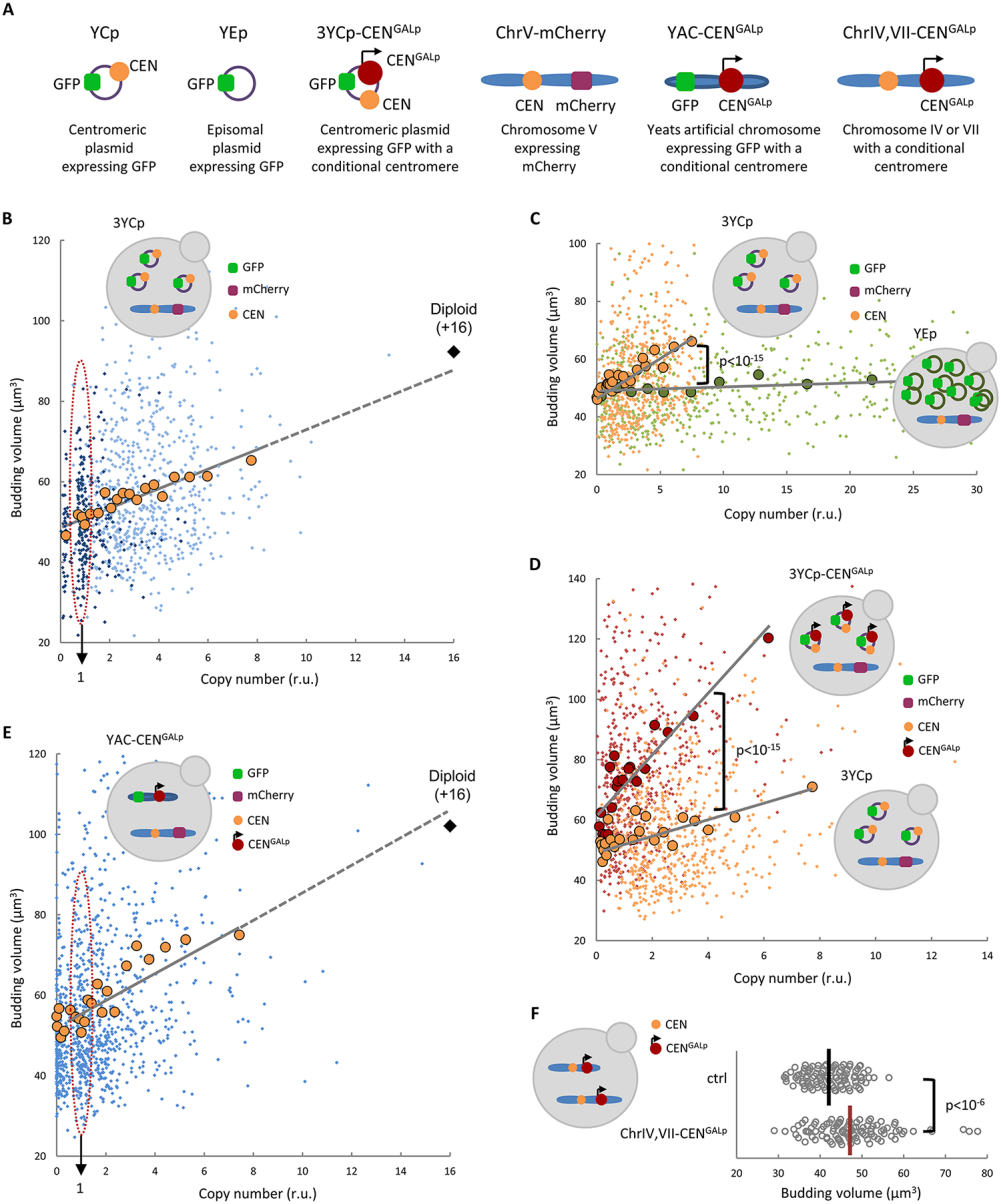
CEN number effects on cell size. (**A**) Scheme showing the different approaches used to assess and manipulate CEN number. (**B**) Yeast cells endogenously expressing mCherry were transformed with one (small purple dots) or three (small blue dots) GFP-expressing YCp vectors, and cell size at budding was determined as a function of vector copy number (GFP/mCherry ratio). Individual budding volumes were binned, and mean values (large orange circles, *N* = 50) and a regression line are plotted. The mean budding size for wild-type diploid cells (which have 16 additional CENs compared to haploid cells) is also plotted (black diamond). (**C**) Cells carrying YEp (green circles) or YCp (orange circles) vectors were analyzed as in (B) to determine cell size at budding as a function of copy number. (**D**) Cells carrying YCp–CEN^*GALp*^ (red circles) or YCp (orange circles) vectors were analyzed as in (B) to determine cell size at budding as a function of copy number under permissive conditions for the additional conditional CEN^*GALp*^ CEN. (**E**) Cells carrying a YAC–CEN^*GALp*^ artificial chromosome were grown at restrictive conditions for the conditional CEN^*GALp*^ CEN to obtain a wide range of copies per cell, returned to permissive conditions, and analyzed as in (B) to determine cell size at budding as a function of copy number. Individual budding volumes (small blue dots) were binned, and mean values (large orange circles, *N* = 50) and a regression line are plotted. The mean budding size for wild-type diploid cells is also plotted (black diamond). (**F**) Newborn daughter cells carrying additional conditional CEN^*GALp*^ CENs in chromosomes 4 and 7 were grown under permissive conditions until they entered the cell cycle. Individual budding volumes (*N* = 100) and median values are plotted. Correlation analysis and pairwise comparisons were performed with nonparametric tests as described in Materials and methods. Underlying data can be found in S1 Data. CEN, centromere; GFP, green fluorescent protein; YCp, yeast centromeric plasmid; YEp, yeast episomal plasmid.

In control experiments in which the size of yeast cells was carefully measured, we had previously observed that the presence of an empty yeast centromeric plasmid (YCp) produced a slightly larger volume at budding. Interestingly, this effect was exacerbated by increasing the number of empty centromeric vectors with different auxotrophic markers, suggesting that G1 length could be modulated by a genetic determinant present in these extrachromosomal DNA molecules. After ruling out possible effects due to plasmid-borne auxotrophic markers (S1A Fig), we analyzed newborn daughter cells during cell cycle entry in time-lapse experiments and found that, while initial volume was very similar, YCp caused a strong delay in G1 and a larger cell size at budding (S1B and S1C Fig). To assess the effects of YCp copy number at the single-cell level, we inserted a *TEF1p*-driven transcription unit expressing green fluorescent protein (GFP) in YCp vectors and mCherry in chromosome 5 and used different approaches to increase the number of centromeric sequences in the cell, some of them in a conditional manner (Fig 1A). We first analyzed cells in the simplest scenario, i.e., containing three GFP-expressing YCp vectors. Budding volume of control cells displayed a large variability [21,28] but steadily increased with the GFP/mCherry ratios (Fig 1B, see S1 Data for a detailed statistical analysis). Intriguingly, the observed trend was compatible with the doubling in budding volume displayed by diploid cells. A yeast episomal plasmid (YEp), which is present at much higher copy numbers, did not significantly alter budding volume (Fig 1C), thus pointing to the autonomous-replicating sequence (ARS) or the CEN as the YCp-specific genetic determinants modulating cell size at budding. To discern between these possibilities, we used a yeast CEN placed immediately downstream from the inducible *GAL1* promoter as a conditional CEN that, by growing cells under conditions that activate (galactose) or repress (glucose) transcription from the *GAL1* promoter, can be switched off or on, respectively [29]. We introduced this conditional CEN (Fig 1A) into three different YCp vectors and observed that, under permissive conditions, cell volume at budding increased with a much steeper slope compared to unmodified YCp (Fig 1D). To rule out possible topological effects due to the circular conformation of YCp vectors, we used a linear yeast artificial chromosome (YAC) containing a conditional CEN to obtain a wide range of copy numbers per cell. As shown in Fig 1E, budding volume correlated with YAC copy number in a similar manner to that obtained with YCp vectors. Moreover, as this effect was also observed with a circular YAC derivative (S2 Fig), we were able to rule out possible additional effects of telomeric sequences. Finally, introducing conditional CENs into chromosomes 4 and 7 caused a significant increase in the budding volume of newborn daughter cells obtained by differential gradient centrifugation when allowed to enter the cell cycle under permissive conditions (Fig 1F). As previously described [30,31], high copies of centromeric vectors caused a short mitotic delay (S3A Fig) that depended on the spindle-assembly checkpoint (SAC) [32,33]. However, this delay was much shorter than that observed during cell-cycle entry (S1C Fig), suggesting that elevated CEN copies have a greater impact in G1. Accordingly, additional CEN sequences caused a small but significant increase in the proportion of cells in G1 phase in asynchronous cultures (S3B Fig). Overall, these data indicate that CEN number modulates G1 length in daughter cells and regulates their size at budding.

**Fig 2:**
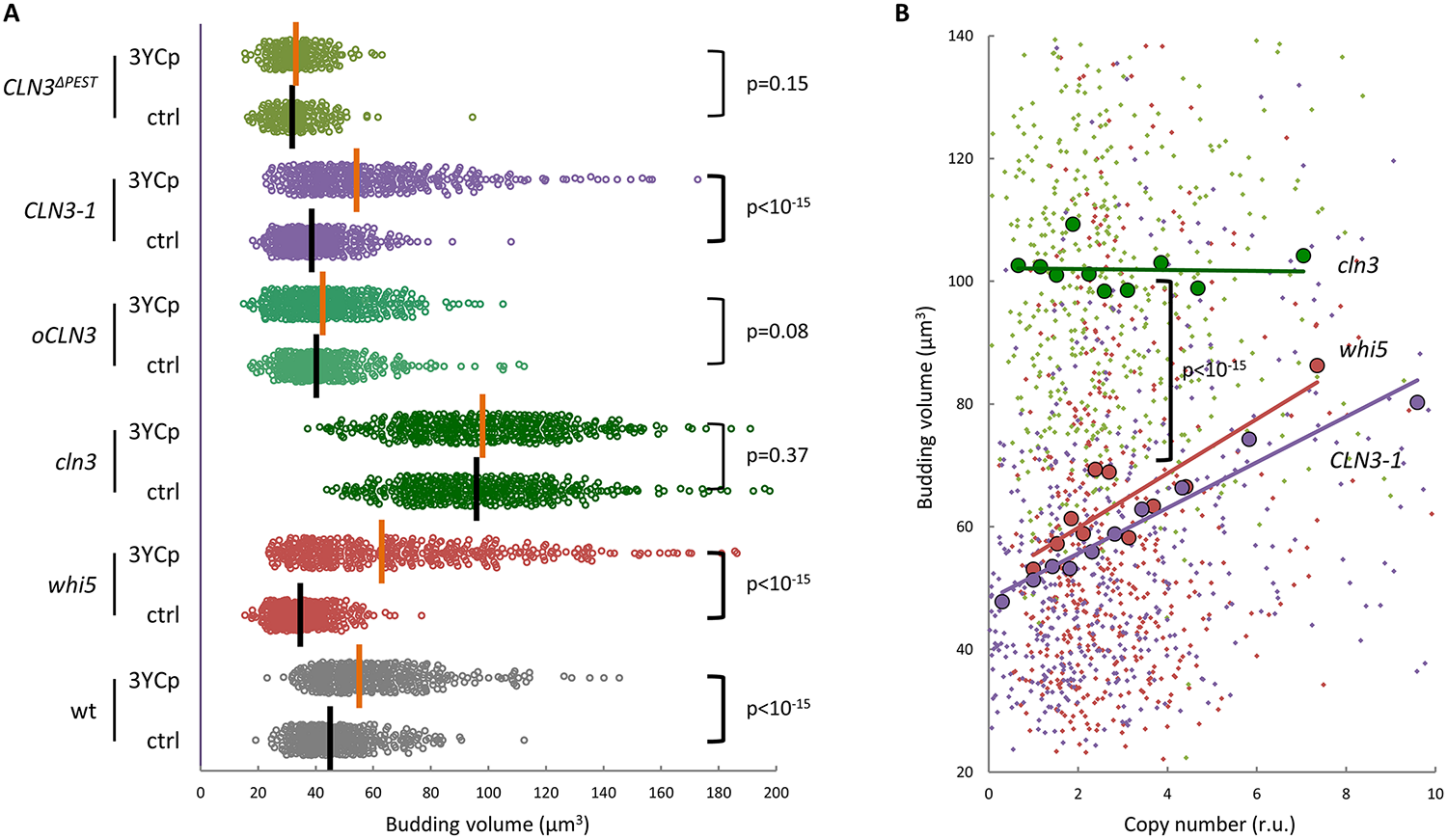
Exceeding CENs modulate cell size in a Cln3-dependent manner. (**A**) Cells with the indicated genotypes carrying three YCp vectors (3YCp) or none (ctrl) were analyzed to determine cell size at budding. Individual data (*N* > 400), and median values are plotted. (**B**) Cells with the indicated genotypes carrying three YCp vectors were analyzed as in Fig 1B to determine cell size at budding as a function of copy number. Individual budding volumes (small dots) were binned, and mean values (large circles, *N* = 50) and a regression line are plotted. Correlation analysis and pairwise comparisons were performed with nonparametric tests as described in Materials and methods. Underlying data can be found in S1 Data. CEN, centromere; YCp, yeast centromeric plasmid.

Budding yeast cells mainly determine their size at Start [4]. Thus, we reasoned that signals originating from the CEN could target specific components of the Start network. YCp vectors clearly increased budding volume in cells deficient in Whi5 (Fig 2A and 2B), thus ruling out this transcriptional repressor of the G1/S regulon [34,35]. By contrast, cells lacking Cln3, the most upstream G1 cyclin [8,10,36] acting at Start, did not increase their size further, indicating that Cln3 is essential in the mechanisms that allow centromeric signals to modulate cell size. Overexpression of wild-type Cln3, which causes a strong nuclear accumulation of this G1 cyclin [15,37], also suppressed the YCp-mediated effects on budding size. However, a Cln3–1 hyperstable mutant that also reaches high levels but lacks the C-terminal nuclear-localization signal (NLS) that is essential for nuclear import of Cln3 [38] was as sensitive as wild type to the presence of YCp (Fig 2A and 2B). Supporting the notion that centromeric-dependent effects take place in the nucleus, a different hyperstable Cln3^ΔPEST^ mutant protein that retains the C-terminal NLS and strongly accumulates in the nucleus [15,37] fully suppressed YCp-mediated effects in cell size. Together, our data point to the idea that centromeric-dependent signals target, directly or indirectly, the yeast G1 cyclin in the nucleus.

**Fig 3:**
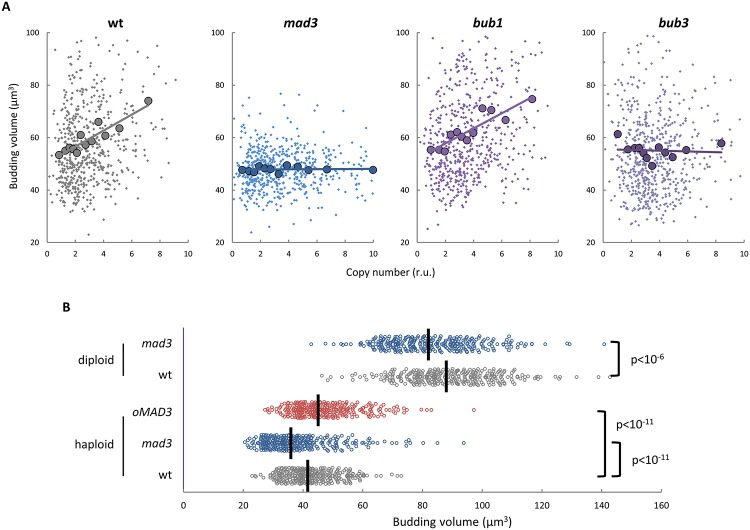
Exceeding CENs require centromeric Mad3/Bub3 signaling proteins to modulate cell size. (**A**) Cells with the indicated genotypes carrying three YCp vectors were analyzed as in Fig 1B to determine cell size at budding as a function of copy number. Individual budding volumes (small dots) were binned, and mean values (large circles, *N* = 50) and a regression line are plotted. (**B**) Newborn daughter cells with the indicated genotypes were analyzed to determine cell size at budding. Mad3 overexpression (*oMAD3*) was attained by inducing a *GAL1p*–*MAD3* construct with 1 mM estradiol in newborn cells expressing the Gal4–hER–VP16 transactivator. Individual data (*N* > 300) and median values are plotted. Correlation analysis and pairwise comparisons were performed with nonparametric tests as described in Materials and methods. Underlying data can be found in S1 Data. CEN, centromere; YCp, yeast centromeric plasmid.

A high-throughput two-hybrid analysis in budding yeast [39] had revealed an interaction between the Cln3 cyclin and Mad3, a component of the kinetochore-signaling network involved in the SAC [40,41]. Thus, we tested whether centromeric signaling proteins could have a role in modulating budding size as a function of YCp copy number (Fig 3A). The budding size of cells lacking either Mad3 or Bub3 was absolutely refractory to increasing copies of YCp while, contrarily, kinase Bub1 did not have any effect. These results suggest that Mad3/Bub3 inhibit Cln3 function in a Bub1-independent manner, thus defining a mechanism different to that executing the SAC.

Newborn daughter haploid and diploid cells lacking Mad3 displayed a smaller volume at budding compared to wild type (Fig 3B). However, size reduction was only moderate compared to the difference between haploid and diploid wild type, and *mad3* cells reduced their size normally from diploid to haploid status. These data suggest that either Mad3 is not required per se in the sensing mechanism or cells must have additional or backup mechanisms to adjust cell size to ploidy (see below). Although Mad3 could not be overexpressed to much higher levels compared to the endogenous copy (S4A and S4B Fig), budding size displayed a clear increase under these mild overexpression conditions (Fig 3B). Considered together, these data reinforce the notion of an inhibitory role for Mad3 in cell cycle entry and cell size determination at budding.

**Fig 4:**
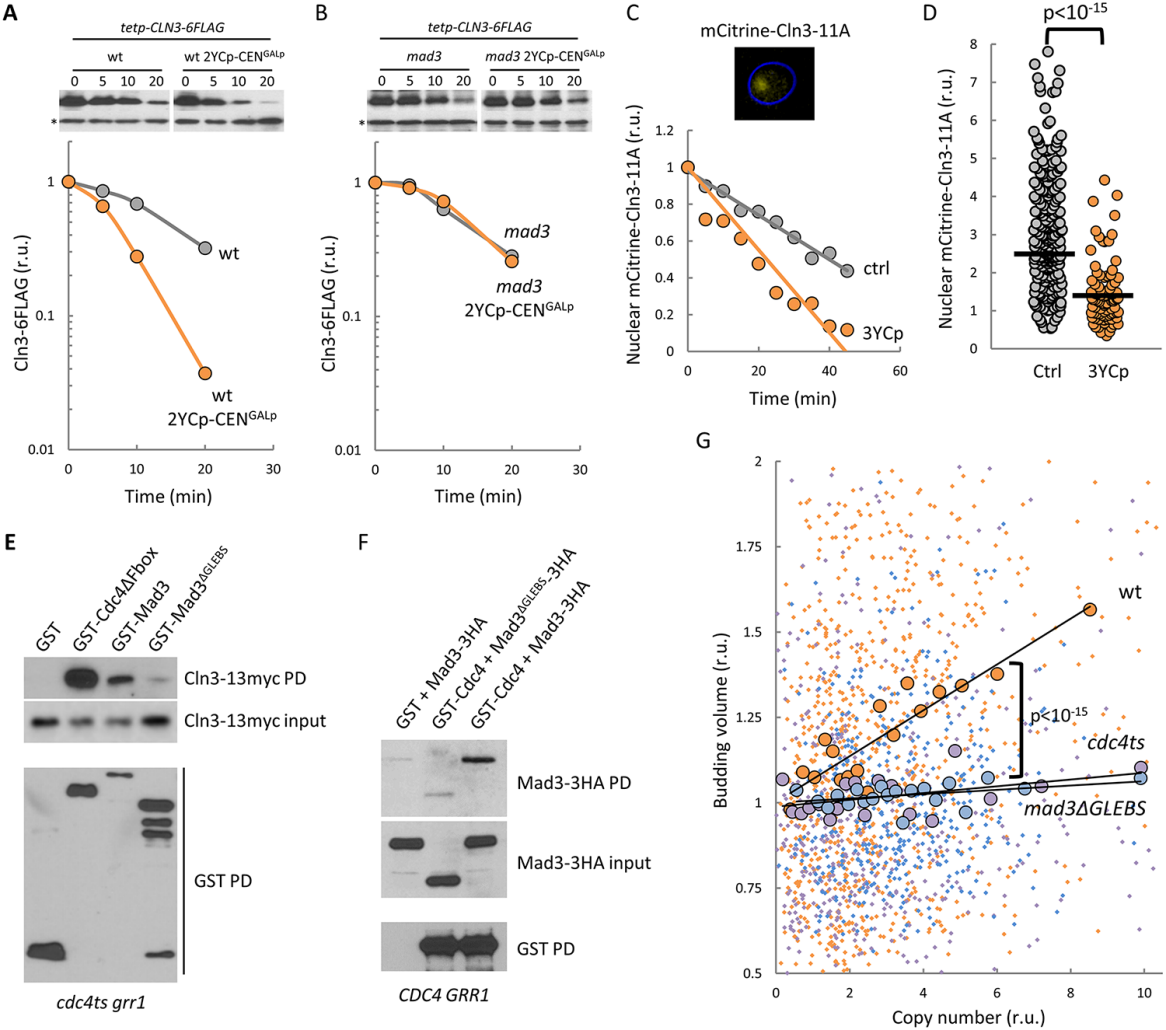
Degradation of cyclin Cln3 by exceeding CENs: Mad3 physical and functional interactions with SCF. (**A**) Analysis of Cln3 stability by promoter shut-off experiments in the presence (orange circles) or absence (gray circles) of two YCp–CEN^*GALp*^ vectors in wild-type cells grown under permissive conditions. After tetracycline addition, cells were collected at the indicated times, and obtained Cln3–6FLAG levels are plotted relative to an unspecific cross-reacting band (asterisk) used as loading control. (**B**) Analysis of Cln3 stability in Mad3-deficient cells as in (A). (**C**) Analysis of mCitrine–Cln3–11A stability by time-lapse microscopy in the presence (orange circles) or absence (gray circles) of three YCp vectors. Nuclear levels of mCitrine–Cln3–11A in cells were determined at the indicated times after cycloheximide addition, and mean values (*N* = 100) are plotted. (**D**) Analysis of mCitrine–Cln3–11A accumulation in the nucleus in the presence (orange circles) or absence (gray circles) of three YCp vectors. Nuclear levels of mCitrine–Cln3–11A were determined in G1 daughter cells with 50–60 μm^3^ of volume. Individual data (*N* = 90) and median values are plotted. (**E**) Cell extracts (input) and GST PDs of *cdc4ts grr1* cells expressing Cln3–13myc and GST fusions to Cdc4ΔFbox, Mad3, or Mad3ΔGLEBS were analyzed by immunoblotting with either αmyc (top panels) or αGST (bottom panel) antibodies. (**F**) Cell extracts (input) and GST PDs of cells expressing Mad3–3HA or Mad3 ΔGLEBS–3HA and either GST or GST–Cdc4 were analyzed by immunoblotting with either αHA (top panels) or αGST (bottom panel) antibodies. (**G**) Cells with the indicated genotypes carrying three YCp vectors were analyzed as in Fig 1B to determine cell size at budding as a function of copy number. Individual budding volumes (small dots) were binned, and mean values (large circles, *N* = 50) and a regression line are plotted. Correlation analysis and pairwise comparisons were performed with nonparametric tests as described in Materials and methods. Underlying data can be found in S1 Data. GST, glutathione S-transferase; PD, pulldown; YCp, yeast centromeric plasmid.

Mad proteins use different but complementary mechanisms to modulate degradation of Cdc20 targets by the anaphase-promoting complex (APC/Cdc20), including mitotic cyclins [32,33], which suggests that the Mad3-dependent effects of YCp vectors on budding volume could be mediated by degradation of Cln3. Supporting this idea, Skp1 is a highly expressed centromeric protein that is also present in SCF, the E3 ubiquitin ligase required to degrade Cln3 [18]. We found that the presence of YCp vectors strongly increased the degradation rate of Cln3 in promoter shut-off experiments, and more importantly, this effect required Mad3 (Fig 4A, 4B, S5A and S5B Fig). To support these findings further, we used a partially hyperstable and hypoactive mutant (Cln3–11A) fused to mCitrine that has no gross effects on cell cycle progression [42] but allows detection of this cyclin in G1 cells by fluorescence microscopy to monitor Cln3 degradation specifically in the nucleus. Although cells expressing mCitrine–Cln3–11A displayed an increased volume at budding when compared to wild-type cells, the presence of YCp vectors caused a similar relative increment in their budding size (S6A Fig), which validated its use. Notably, by measuring mCitrine–Cln3–11A levels in G1 cells after cycloheximide addition, we found that the presence of YCp vectors also increased the degradation rate of this G1 cyclin in the nucleus in a Mad3-dependent manner (Fig 4C and S6B Fig). Accordingly, mCitrine–Cln3–11A steady-state levels were strongly decreased by YCp in the nucleus of G1 cells within the same volume range (Fig 4D). Since Cln3 is rate-limiting for triggering Start and setting the critical size at budding, these results would explain why the presence of YCp vectors causes a larger cell size. Next we analyzed the interaction between Mad3 and Cln3 by affinity purification and found that they yielded relative coprecipitation efficiencies similar to Cln3 and Cdc4ΔFbox (Fig 4E), the adaptor protein that recruits Cln3 to SCF in the nucleus [18]. Interestingly, we were able to detect an interaction between Cdc4 and Mad3 (Fig 4F), which suggests that Mad3 is present with Cdc4 in SCF complexes. Mad3 contains a GLEBS domain that is known to interact with Bub3 and, as a likely consequence, with Skp1 [43], and we found that Mad3 lacking the GLEBS domain does not efficiently interact with either Cdc4 or Cln3 (Fig 4E and 4F). Finally, modulation of budding size as a function of YCp copy number was strongly dampened by deleterious SCF mutations or deletion of the Mad3 GLEBS domain (Fig 4G and S7 Fig), supporting the essential role of a SCF–Cdc4/Mad3 complex in boosting Cln3 degradation to modulate cell size at budding as a function of CEN copy number.

**Fig 5:**
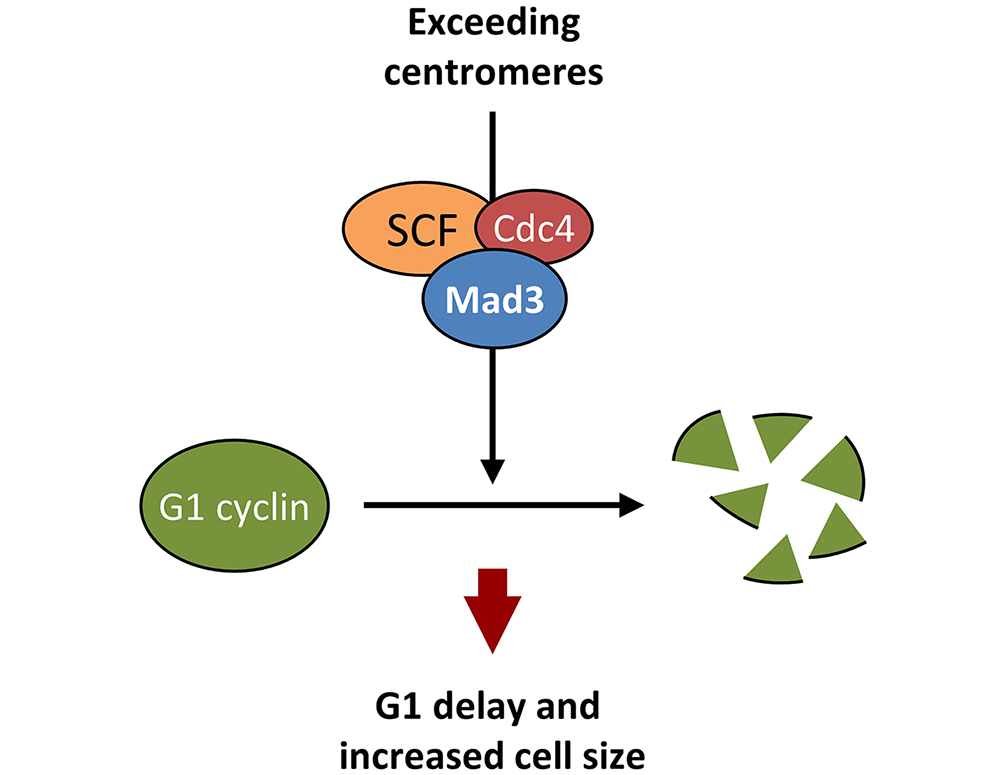
CEN signaling proteins cooperate with SCF–Cdc4 to enhance degradation of the yeast G1 cyclin and modulate cell size in budding yeast. When present in excess, centromeres accelerate Cln3 degradation in the nucleus with the essential participation of Mad3, a centromeric signaling protein that interacts with Cdc4 and requires SCF function to modulate cell size as a function of centromere number. CEN, centromere.

In summary, we have uncovered a pathway that links centromeric signaling proteins to G1 cyclin stability and, hence, cell size determination in budding yeast (Fig 5). SCF–Cdc4 is estimated to be at low levels in the nucleus of yeast cells, and we envisage that Mad3, which is present at much higher levels, could act as a co-adaptor to increase the affinity of Cdc4 for Cln3. Strikingly, Cdc4 and Cdc20 display a high degree of similarity (34.2%) and contain WD40 segments that are used to interact with client proteins. However, the interaction of Mad3 would have different outcomes: (1) prevent Cdc20 from binding its targets in metaphase and (2) acting as an adaptor bridging Cln3 to Cdc4 in G1.

Mad3 is present at rather constant levels throughout the cell cycle [41], and Spc105, a scaffold protein involved in Mad3 activation at kinetochores by SAC [32], is already present in CENs in G1 [44]. Thus, by mechanisms different from those operating the SAC, Mad3 could be specifically activated in G1 at the kinetochore and sustain degradation of Cln3 at levels proportional to the number of CENs during G1 progression. Alternatively, we would like to speculate that the pathway uncovered here could belong to a Mad3-dependent checkpoint triggered by the excess of a kinetochore component that, being synthesized as a function of cell mass, would act as ploidy-mass reporter. Since Mad3-deficient or overexpressing cells do not display strong alterations in cell size, Mad3 would have a role as an effector of the checkpoint, not as sensor. Furthermore, the uncovered mechanism could be used to ensure that CENs congregate at the spindle-pole body (SPB) [45] before cell-cycle entry in budding yeast. While structural determinants of centromeric DNA are strikingly different in yeast and mammalian cells, kinetochore structural and signaling proteins are very well conserved. For this reason, we envisage that the mechanism operating in budding yeast could also exist across the evolutionary scale.

Previously proposed mechanisms to adjust cell size to ploidy [3] have not received sufficient experimental support. Although Whi5 is expressed at levels that depend on ploidy [42], diploid cells lacking one *WHI5* copy are larger than haploid wild-type cells. On the other hand, introduction of additional Cln3-targeted promoters delays cell-cycle entry and increases cell size at budding [46]. However, it remains unclear whether titration of Cln3 by genome duplication is sufficient to produce a diploid cell size. We propose that, most likely with the contribution of these mechanisms, CEN-dependent degradation of Cln3 may play a pivotal role in scaling size with ploidy, a universal property of cells.

## Materials and methods

### Growth conditions and strain constructions

Cells were grown in SC medium with 2% glucose at 30 °C unless stated otherwise. Late G1-arrested cells were obtained by treating exponential cultures at OD_600_ = 0.5 with 5 μg/ml α factor for 105 min at 30 °C. Conditional CEN^*GALp*^ CENs were inhibited by addition of 2% galactose to culture medium. Cycloheximide was added at 25 μg/ml to inhibit protein synthesis. Cln3–3HA half-life was analyzed in *tet*-promoter shut-off experiments by adding tetracycline to 1 μg/ml [47]. *MAD3* overexpression was attained by inducing a *GAL1p-MAD3* construct with 1 mM estradiol in cells expressing the Gal4–hER–VP16 transactivator [48]. Yeast parental strains and methods used for chromosomal gene transplacement and PCR-based directed mutagenesis have been described [37]. Centromeric plasmids and yeast artificial chromosomes were obtained by multiple-fragment recombination [49] in yeast cells. Conditional CEN^*GALp*^ CENs (*GAL10p–CEN4*) were inserted in chromosomes 4 and 7 by CRISPR/Cas9-driven recombination [50]. The ΔGLEBS mutant of Mad3 lacked the C-terminal 155 amino acids. Cln3–1 [11] and Cln3^ΔPEST^ [15] are both hyperstable mutant proteins, but only Cln3^ΔPEST^ retains the C-terminal NLS [38]. The Cln3–11A mutant protein is a hypoactive and hyperstable cyclin that contains 11 amino acid substitutions (R108A, T420A, S449A, T455A, S462A, S464A, S468A, T478A, S514A, T517A, T520A) [42]. The Cdc4ΔFbox protein has been already described [18].

### Time-lapse microscopy

Yeast cells were analyzed by time-lapse microscopy in 35-mm glass-bottom culture dishes (GWST-3522, WillCo) essentially as described [28] using a fully-motorized Leica AF7000 microscope. Time-lapse images were analyzed with the aid of BudJ, an ImageJ (Wayne Rasband, NIH) plugin that can be obtained from www.ibmb.csic.es\home\maldea to obtain cell dimensions and fluorescence levels in cellular and nuclear compartments [28]. Briefly, cell boundaries are detected as pixels markedly darker compared to both the surrounding background and the cell interior. Once outliers have been removed, an ellipse is fitted to the obtained boundary pixel array, and major and minor axes are used to calculate the cell volume assuming a prolate as shape. The same cell is followed through consecutive time-lapse images by using the center of the ellipse as seed point to obtain radial profiles in the following image.

### Affinity purification and immunoblotting

GST-tagged proteins were affinity purified with glutathione beads (GE Healthcare) from cell extracts as described [37]. Immunoblot analysis [51] was performed with antibodies against HA (12CA5, Roche), FLAG (M2, Sigma), myc (9E10, Sigma), and GST (polyclonal, Millipore).

### Miscellaneous

Small daughter cells were isolated from Ficoll gradients as described [52]. DNA content distributions were obtained by Fluorescence Activated Cell Sorting [51].

### Statistical tests

Pairwise comparisons were performed with non-parametric tests. Specifically, median cell volumes at budding were compared with a Mann–Whitney U test. On the other hand, correlation of cell volume at budding with GFP/mCherry ratios was analyzed with a Spearman rank test. For pairwise analysis, data were subject to bootstrap resampling (*N* = 100), and the resulting median slopes were compared by a Mann–Whitney U test. For both median and regression analysis, the resulting *p* values are shown in the corresponding figure panels.

## Supporting information

S1 Fig CEN number effects in G1 phase. (**A**) Newborn daughter wild-type cells with three YCp vectors (3YCp) or none (ctrl) or from a prototrophic *URA3 LEU2 TRP1* derivative (3AUX) were analyzed to determine cell size at budding. Individual data (*N* > 300) and median values (vertical lines) are plotted. (**B**) Newborn daughter cells with three YCp vectors (3YCp) or none (ctrl) were analyzed by time-lapse microscopy to determine initial and budding volumes. Individual data (*N* > 90) and median values (vertical lines) are plotted. (**C**) G1 lengths corresponding to cells analyzed in panel B. Individual data (*N* > 90) and median values (horizontal lines) are plotted. Pairwise comparisons were performed with non-parametric tests as described in Materials and methods. Underlying data can be found in S1 Data. CEN, centromere; YCp, yeast centromeric plasmid.
(TIF)

S2 Fig CEN number effects by a conditional-centromeric circular chromosome on cell size. Cells carrying a YAC–CEN^*GALp*^ artificial circular chromosome with no telomeric sequences were grown at restrictive conditions for the conditional CEN^*GALp*^ CEN to obtain a wide range of copies per cell, returned to permissive conditions and analyzed as in Fig 1B to determine cell size at budding as a function of copy number. Individual budding volumes (small gray dots) were binned, and mean values (large orange circles, *N* = 50) and a regression line are plotted. The mean budding size for wild-type diploid cells is also plotted (black diamond). Nonparametric correlation analysis was performed as described in Materials and methods. Underlying data can be found in S1 Data. CEN, centromere.
(TIF)

S3 Fig CEN number effects in G2/M phases. (**A**) Wild-type or Mad3-deficient cells with three YCp vectors (3YCp) or none (ctrl) were arrested in late G1 with α factor and released into fresh medium to determine the percentage of binucleate cells at the indicated times. (**B**) DNA content distributions of wild-type cells carrying the indicated vectors or none (ctrl) under permissive conditions for CEN^*GALp*^ CENs. Bars at the top correspond to the respective percentage of G1 cells in each sample. Underlying data can be found in S1 Data. CEN, centromere; YCp, yeast centromeric plasmid.
(TIF)

S4 Fig Overexpression of *MAD3* under the *GAL1* promoter. (**A**) Immunoblot analysis of *GAL1p*-driven Mad3–6FLAG levels at different times after *GAL1p* induction with 1 mM estradiol. Extracts from cells expressing Mad3–6FLAG at endogenous levels and untagged cells were also loaded as reference. A Coomassie Blue–stained major band is shown as loading control. (**B**) Quantification of Mad3–6FLAG levels shown in panel (A). Underlying data can be found in S1 Data.
(TIF)

S5 Fig Degradation of cyclin Cln3 by exceeding CENs. (**A**) Analysis of Cln3 stability by promoter shut-off experiments in the presence (orange circles) or absence (gray circles) of two YCp–CEN^*GALp*^ vectors in wild-type cells grown under permissive conditions. After tetracycline addition, cells were collected at the indicated times, and obtained Cln3–6FLAG levels are plotted relative to an unspecific cross-reacting band (asterisk) used as loading control. (**B**) Analysis of Cln3 stability in Mad3-deficient cells as in (A). Underlying data can be found in S1 Data. CEN, centromere; YCp, yeast centromeric plasmid.
(TIF)

S6 Fig YC effects on mCitrine–Cln3–11A and stability in Mad3-deficient cells. (**A**) Cells expressing mCitrine–Cln3–11A carrying three YCp vectors (3YCp) or none (ctrl) were analyzed to determine cell size at budding. Individual data (*N* > 400) and median values (vertical lines) are plotted. Pairwise comparisons were performed with a nonparametric method as described in Materials and methods. (**B**) Analysis of mCitrine–Cln3–11A stability in Mad3-deficient cells. Nuclear levels of mCitrine–Cln3–11A were determined by time-lapse microscopy in *mad3* cells and in the presence (orange circles) or absence (gray circles) of three YCp vectors after cycloheximide addition as in Fig 4C. Mean values obtained from individual cells (*N* = 100) are plotted. Underlying data can be found in S1 Data. YCp, yeast centromeric plasmid.
(TIF)

S7 Fig Cell size effects by exceeding CENs in SCF-deficient cells. Cells with the indicated genotypes carrying three YCp vectors were analyzed as in Fig 1B at the restrictive temperature for *cdc53ts* and *cdc34ts* alleles to determine cell size at budding as a function of copy number. Individual budding volumes (small dots) were binned, and mean values (large circles, *N* = 50) and a regression line are plotted. Correlation pairwise comparisons were performed with a nonparametric test as described in Materials and methods. Underlying data can be found in S1 Data. CEN, centromere; YCp, yeast centromeric plasmid.
(TIF)

S1 Data Source data for all plots in manuscript. (XLSX)

## Abbreviations

APC: anaphase-promoting complex
ARS: autonomous-replicating sequence
CEN: centromere
GFP: green fluorescent protein
GST: glutathione S-transferase
NLS: nuclear localization signal
PD: pulldown
SAC: spindle-assembly checkpoint
SCF: Skp1/Cul1/F-box complex
SPB: spindle-pole body
YAC: yeast artificial chromosome
YCp: yeast centromeric plasmid
YEp: yeast episomal plasmid
